# Strategic predictors of performance in a divided attention task

**DOI:** 10.1371/journal.pone.0195131

**Published:** 2018-04-05

**Authors:** Róbert Adrian Rill, Kinga Bettina Faragó, András Lőrincz

**Affiliations:** 1 Faculty of Informatics, Eötvös Loránd University, Budapest, Hungary; 2 Faculty of Mathematics and Computer Science, Babeş-Bolyai University, Cluj-Napoca, Romania; University of British Columbia, CANADA

## Abstract

In this study we investigate the strategies of subjects in a complex divided attention task. We conducted a series of experiments with ten participants and evaluated their performance. After an extensive analysis, we identified four strategic measures that justify the achievement of the participants, by highlighting the individual differences and predicting performance in a regression analysis using generalized estimating equations. Selecting the more urgent task and user action between multiple simultaneous possibilities form two of the strategic decisions, respectively. The third one refers to choosing a response within the same task when the opportunity is present. The fourth and most important measure of strategy involves thinking ahead and executing an action before a situation would become critical. This latter one has the effect of reducing later cognitive load or timing constraints and it is shown to explain almost as much variance in performance as the other three, more straightforward predictors together. In addition to determining these strategic predictors, we also show how manipulating task difficulty induces a shift in strategy, thus impairing human performance in the rehearsed task. The results of this study indicate that considerable differences in the divided attention ability of normal subjects can be identified early and with simple measurements. The importance of describing and analyzing strategies is also emphasized, which can substantially influence performance in complex tasks and may serve training needs.

## Introduction

In 1651 philosopher Thomas Hobbes introduced the term *train of thoughts* in his famous Leviathan [1]: “By consequence, or train of thoughts, I understand that succession of one thought to another, which is called, to distinguish it from discourse in words, mental discourse. When a man thinketh on anything whatsoever, his next thought after is not altogether so casual as it seems to be. Not every thought to every thought succeeds indifferently.”

The meaning today is somewhat different. Train of thought refers to the sequence of ideas and the interconnection between them expressed during a coherent discourse. It is the process of linking one thought after another to form a concise path of reasoning.

Relating to this concept, *Train of Thought* is an Attention game of Lumosity (http://www.lumosity.com/), an online platform for training five core cognitive abilities: attention, processing speed, memory, flexibility and problem solving. Lumosity is comprised of a set of computerized games designed by scientists, each aiming to train one cognitive skill [2]. The effectiveness of this online training program in enhancing cognitive abilities has been demonstrated by large-scale studies (see, e.g., [3]). On the other hand, there is a growing body of research that calls the benefits of cognitive training into question. For example, Simons et al. [4] provide a comprehensive examination of the “brain-training” literature, and find little evidence that training improves everyday cognitive performance.

The purpose of the Train of Thought game is to test one’s visual divided attention and working memory by directing continuously oncoming trains to their color matching destination. The direction of the tracks and the path of the moving trains can be altered through flipping switches with mouse clicks. The task is challenging, because the trains are constantly moving and the players are required to focus on multiple targets simultaneously. As performance improves the number of destinations is increased raising this way the difficulty level.

Divided attention refers to an ability of processing multiple sources of information that allows us to carry out more than one task in the same time. It is present in our everyday lives, e.g., when driving a car, shopping, studying, and it is also required by a variety of jobs, such as call center agents, pilots, physicians, engineers. Although it may cause interference and reduce efficiency and accuracy [5], being able to successfully divide attention between multiple activities is important in our daily lives. Divided attention works very differently for people with autism spectrum disorders [6], dyslexic individuals (see, e.g., [7] and the references therein) and persons with schizophrenia [8, 9] among many others. In this work we assess the skills of normal subjects and highlight particular differences through a series of experiments.

Similar to the Train of Thought game we have designed and implemented our own version to challenge the divided attention and working memory of individuals. Players are required to continuously monitor simultaneous tasks, to switch frequently between them, keeping track of each one in order to maximize performance. Often at the same time multiple switch flips are acceptable and the user has to choose the right strategy in order to reach high scores. In the following, our version of Train of Thought will be referred to as the *Divided Attention Game*, or shortly *DA Game*.

The DA Game is a complex task, comparable to multitasking environments. There is no general agreement on the definition of the term multitasking [10]. Some researchers have defined it simply as carrying out two or more tasks at the same time [11], others as a means to accomplish *multiple goals* within the same time period by frequently *switching* between the individual tasks [12, 13]. Other terms which have been frequently used is task switching [14] or dual-tasking [15]. The expression *media multitasking* is differentiated as well, and it is the focus of several studies [16–19]. Multitasking can also refer to the ability itself, of switching between multiple tasks, requiring *conscious shifts* of attention over a short time span [20–22].

It is not our aim to properly classify the DA Game as a particular multitasking environment; it seems to incorporate more than a single component. However, it is definitely different from dual-tasking; individuals have to monitor multiple tasks simultaneously and switch frequently between them for better performance. On the other hand, the individual tasks are equivalent in the sense that they require the same cognitive abilities. In spite of all these, we think that studies conducting experiments and investigating performance in multitasking, or complex environments, are close to our work, especially the ones that also analyze strategies of the participants.

Our work consists of designing, performing and evaluating a set of experiments, in which participants were asked to play with the DA Game over a several day period. The main purpose of the evaluations was to identify strategic performance measures, in order to highlight the individual differences between the participants and to characterize their strategic decisions that predict performance. The difficulty of the DA Game (and Train of Thought too) is determined by several aspects. These are elaborated later in the paper, in the Methods section. For now we mention that we made efforts to decrease the number of these variables in order to reduce complexity as much as possible. We kept one parameter to be able to control task demands. The purpose of the difficulty manipulation is twofold. We have increased the level of difficulty gradually in order to keep the task challenging as players are progressing. In addition, we wanted to test the effect of stress on decision making performance and strategy by means of increasing the frequency of user actions. Furthermore, our explicit goal was to determine such strategic decisions that remain consistent predictors of performance despite the changes in difficulty. Our efforts indicate that such hidden variables can be identified. The results of this study also demonstrate that considerable differences in the divided attention ability of normal subjects can be detected already after a short period of practice. We will address the limitations and the advantages of our studies at the end of the Discussion section. Our significant results give rise to novel questions and point to further experiments.

The paper is organized as follows. First, related studies are reviewed that investigated performance in various complex tasks, with the focus being on the works that were concerned with strategies of participants. This prepares the possibility to frame the relevance of our research question and to state our contributions. Second, we elaborate on the design process of the DA Game, describe the experiments we conducted and define the strategic predictors. Third, the results of the evaluations are presented, illustrating how the strategic measures highlight the individual differences between participants and predict performance in a regression analysis using the generalized estimating equation (GEE) method. Fourth, the results are discussed and the importance of the determined strategic decisions is detailed, relating them to similar strategies from other studies. Finally, we conclude the paper by summarizing our work.

## Related work

A range of studies have been concerned with multitasking ability and examined human performance in complex environments. These works have considered mostly ability constructs and personality traits as predictors of achievement, using various problem solving tasks. In this study we investigate strategic predictors of performance. We start by mentioning that there is no universally agreed-upon definition of strategy. It has been defined as a method used for problem-solving [23], or can be understood as “an approach to engaging the cognitive system to accomplish a goal when other approaches are possible” (see, e.g., [24]). König, Bühner, and Mürling [13] argued that even multitasking can be considered a strategy for effective time-management, however, only for people with large working memory capacity. The importance of strategies in the analysis of performance was emphasized by several works (see, e.g., [23] and the references therein). In the following, we mention studies that are concerned with ability and personality constructs as predictors of performance, and then turn our attention to strategies investigated in a variety of multitasking settings.

### Ability constructs as predictors

One of the most studied ability constructs in relation to multitasking, is the skill of attention. Arthur et al. [25] considered performance in a computer-controlled game-like task, called Space Fortress. The results proved that individual differences in visual attention correlated with task performance, even after taking into account training effects. A different computerized task was used by Szymura and Nȩcka [26] to analyze the connection between visual selective attention and basic personality dimensions by formulating theoretical models. Attention in general was assessed by several other works as well in different tasks (see, e.g., [13, 27, 28]). Intelligence or reasoning has also been considered by a range of studies [13, 20, 23, 26–32].

A synthetic work environment involving four parallel tasks was exploited by several researchers [20, 24, 30, 33–35]. One study showed that cognitive abilities positively correlated with multitasking performance [20], while in [30] it was found that they also predicted error types. Some of these works have investigated the relationship between working memory capacity and performance. For example, Hambrick et al. [24] showed that it is an important factor underlying the ability to multitask. This is in line with the results of König, Bühner, and Mürling [13], who found working memory to be the most important predictor of performance in a different, standardized scenario for multitasking. Working memory has been proven to be a good predictor by a number of other works as well [17, 20, 21, 23, 27, 30, 32, 36, 37].

### Personality traits as predictors

The synthetic work environment mentioned previously was used by some studies to analyze the relationship between task performance and personality variables too. For example, the theoretical models of [26] incorporate basic personality dimensions of individual differences, or in [34] it was found that non-ability factors also underlay success or failure. In this latter work, especially neuroticism predicted performance, a personality variable investigated by others as well [16, 26, 38].

Other non-ability traits analyzed by several studies are Type A behavior pattern [11, 12, 34], need for closure [37, 39], extraversion [13, 16, 26, 34, 38]. Out of these, neuroticism and extraversion are members of the Big Five or OCEAN personality traits, considered by some of the works listed.

One of the most widely assessed individual difference variable in relation with multitasking performance is polychronicity, an individual’s preference for performing multiple tasks at once [11–13, 22, 28, 34, 35, 40]. This is expected to positively correlate with performance, which is the case in the study of Zhang et al. [40]. They found that polychronic people switched between processes more frequently in a dual-process control task and achieved better performance than monochronic subjects. Polychronic individuals performed better in a dual-task setting too [28], by switching more between tasks under time constrained conditions. The results of Sanderson et al. [22] showed that polychronicity played an important moderating role in the relationship between multitasking ability and performance in a variety of jobs. However, other researchers failed to find a significant relation between polychronicity and multitasking performance [11–13, 34].

### Strategic decisions as predictors

The ability and personality constructs mentioned above are a few of the most important examples investigated in the vast literature available and the references are not reviewed in detail, since this is outside the scope of the present work. Here we are concerned with strategies for problem solving, investigated by several works, some of which were mentioned in the previous sections too. In the experiments of Arthur et al. [25] for instance, well documented optimal strategies were conveyed to the subjects. According to the study, these were developed by experienced operators and constitute mostly in prioritizing some actions over other ones (for more details see the cited work and the references therein).

Communicating optimal strategies to subjects is not a common practice in research. Instead scientists manipulate the difficulty or the circumstances of the tasks in their experiments in order to observe changes in decision making, which influence multitasking performance, or to study the difference between contrasting personality types (see, e.g., [26]). Inducing time pressure is one widely used method for modifying task demands [29, 41], which is closely related to time sharing ability [42]. Timing or prioritizing tasks is another aspect that affects performance in multitasking settings [24, 33, 42]. Task preference can be influenced through altering payoffs [33, 43]. To maximize performance when performing multiple tasks in parallel, interleaving between them is essential [43–45]. However, this leads to interruptions [45, 46], which can have negative effects on achievement and also represent an issue from a cognitive modeling perspective [47]. Solving complex problems may also require a general tradeoff of physical and cognitive resources [21, 23]. Particularly, increased cognitive load usually impairs performance, but may also carry benefits [48]. From the perspective of strategies, the referenced works implicitly or explicitly study adaptability of subjects, i.e. how they modify their way of problem solving as a response to changing task demands. On the other hand, some researchers may deliberately manipulate task particularities to force specific, well-defined strategies to work best (see, e.g., [48]). Others may have the focus on identifying and comparing strategies in dynamic tasks [31, 49]. In the following we review the studies mentioned in this paragraph with the focus being on how strategies are defined in the various tasks considered.

Szymura and Nȩcka [26] analyzed the proportion of false alarms to the overall number of errors, interpreted as “a measure of strategy adopted by a subject: impulsive responding versus the reflective preference to misses rather than false alarms”. The data in this study indicated that neuroticism was connected to a speed/accuracy tradeoff strategy, consisting in sacrificing speed for accuracy when the cognitive task was more demanding as opposed to sacrificing accuracy for speed in the easy task condition. Furthermore, in one of their experiments, subjects who allocated their attention equally to both the primary and secondary tasks achieved better performance.

Gonzalez [29] exploited a dynamic decision making task and found that participants under high time constraints performed worse than those under low time constraints, even after receiving three times more practice. She also analyzed strategies, referred to as decision heuristics, which consist in decision making taking into account the particularities of the task. Simple heuristic models were compared to actual decisions of the subjects, and low time constraints and increased practice were associated with a poorer fit. Conversely, severe time constrains and low cognitive abilities with minimal practice showed improved fit, suggesting that more time and cognitive abilities can lead to the acquisition of more complex context-based knowledge, to be used later under time constraints.

Moon and Anderson [41] used a simplified version of the task considered in [25], to analyze time interval estimation. They focused on the effects of memory contamination and time pressure, as mechanisms for the too-early bias, i.e. the tendency to respond too early and underestimate an intermediate target interval. One of their findings was that participants performed significantly better when estimating a target interval in alternation with a short interval, than with a long interval because in the former case one has to meet only an upper limit for the short interval and can use a simple strategy of executing two taps as quickly as possible.

Schumacher et al. [42] investigated time sharing in a dual-task setting involving two choice reaction tasks: an auditory-vocal and a visual-manual task. Their experiments showed that virtually perfect time sharing is possible, i.e. procedural decision making and response selection for multiple tasks can proceed simultaneously. The results also demonstrated that dual-task interference can be modulated by different scheduling strategies of the subjects: a cautious one with minimal temporal overlap in processing of the two tasks might lead to high interference, while a daring one with large processing overlap is consistent with low interference, i.e. virtually perfect time sharing. Controlling tasks simultaneously by switching between them more often is a specific control strategy of polychrons who tend to outperform monochronic individuals [28, 40] in dual-task settings.

The synthetic work environment mentioned in previous sections was assessed by Wang, Proctor and Pick [33] too, who considered payoff effects on strategy development and change. The individual-task payoffs were varied between participants and strategy was defined simply as prioritizing one task over another in order to maximize performance. It was found that the adopted strategies reflected the relative importance of the tasks and when payoffs were changed, the strategies were modified as well. However, the residual effects of initial payoffs were still present, suggesting that payoffs in multitasking need to be explicit and practice is required for achieving maximum performance.

Hambrick et al. [24] showed that working memory predicted the use of an effective strategy. In close relation with the strategy description of Wang, Proctor and Pick [33], strategy was defined as two types of response probabilities that reflect the likelihood of participants to follow a response in one task with a response within the same task, in contrast to a response in a different task. Results suggested that individual differences in strategy use substantially contributed to performance.

The simulation of a real-world scenario was investigated by Janssen and Brumby [44], where participants had to dial a telephone number while driving. The attention interleaving strategies revealed that in order to maximize performance in the driving task, subjects chose to return their attention to steering before the natural subtask boundary in dialing. In a more recent study [43], using a similar typing-while-tracking dual-task, the authors found that interleaving strategy was influenced by change in task characteristics and monetary incentives, together with individual differences in typing ability. A computational modeling analysis assessed in both studies suggested that people adapt their strategies to meet specific performance objectives and to maximize payoff.

Interleaving strategies were tested in a simple office-based task experiment too [45]. The authors of this study provided results showing that interleaving decisions of users were consistent with a strategy of maximizing the marginal rate of return. On the other hand, task interleaving was found to be costly, with interruptions leading to errors in some cases.

Interruptions in multitasking can be quite frequent. Adler and Benbunan-Fich [46] examined self-initiated interruptions in a custom experimental environment with predefined tasks. Participants answered open-ended questions on their reasons for switching between tasks. Some reported a deliberate strategy of focusing at one task at a time. The negative and positive reasons were organized into different categories. The findings indicated that negative feelings triggered more self-interruptions, which in turn degraded performance.

Dealing with external or internal interruptions and task switching in complex environments is a relevant question from a cognitive modeling standpoint too. West and Nagy [47] extend previous theories by addressing the problem of hierarchical task representations in a real-life routine work environment, namely network maintenance and installation in a telecommunications company, to handle unexpected interruptions and opportunistic task switching. They propose to break up unit control structures at points where interruptions are likely, so that the task could be quickly finished or abandoned, assuming this is not constrained by safety and/or technical reasons. The identity of the abandoned and last completed task are stored in memory or in an external artifact, so that work can be resumed later or passed on to someone else. This leads to multitasking and the need of responding to changing demands, by taking into account the constraints and the context of the situation. Consequently, it is important to be able to coordinate tasks and adapt to the situation when unexpected changes occur or goal conflicts arise.

Schunn and Reder [23] demonstrated that individuals differ in strategy adaptivity in a dynamic and complex air traffic control task. The primary strategy measure was defined as a proportion of the times that a participant selected to complete the plane landing task one particular way of all the times that a plane was landed and two choices were available. This strategic decision involved a general tradeoff of physical and cognitive resources. The authors analyzed the defined ratio as a function of whether previous attempts of the task were successful and found evidence for a general adaptivity.

Adaptability in multitasking was considered for example by Morgan et al. [21], who analyzed how participants respond to changing demands in a flight simulator task, in which strategic deployment of attentional resources is critical. The main finding was that multitasking ability and adaptability might be overlapping but separate constructs.

Hoffmann, von Helversen, and Rieskamp [48] were concerned about people’s performance in demanding work environments, considering the cognitive strategies they used in a multiple-cue judgment task. Two strategies were differentiated: a similarity-based strategy which assumes exemplars stored in memory and is more accurate in a nonlinear judgment task, and a more demanding rule-based strategy best suited for linear judgment problems. It was shown that cognitive load can increase performance, by inducing a shift to a similarity-based judgment strategy leading to higher performance.

Strategies were also investigated in a virtual reality setting [49]. The authors in this study characterized three motion patterns, i.e. search strategies, which occurred during spatial navigation in a virtual maze. The results indicated that the observed search strategies are strong predictors of performance changes and the overall level of success in the spatial task. Response patterns as strategies were also studied in a similar dynamic spatial orientation task by Peña et al. [31]. In this work three different ways of solving the task were identified: segmentary, holistic planned and holistic feedback dependent. In the first one participants tend to respond frequently without integrating information, as opposed to the latter two, where individuals respond less either by planning before acting or selecting actions based on the effect of previous ones. According to the authors, these strategies are closely related to those identified by Kallai et al. [49].

It is clear from the above review that tasks and strategies considered by previous studies for testing multitasking performance were different from ours. It is hard to devise a consistent approach, which could dissociate strategies in divided attention tasks. We mention task prioritization as a common way for influencing performance. In this case, manipulations are often explicit; available time or payoffs of the individual tasks are manipulated. In turn, the issue if particular strategic decisions can be identified in any given task is a relevant research topic.

Strategies of subjects may not be the result of careful planning. The general approach that seems to arise is to manipulate difficulty (time pressure, cognitive load, payoff), which causes shifts in strategy and this is what has an impact on performance. Also, identifying strategies and comparing them across subjects is useful for explaining individual differences in performance.

Our contribution consists in analyzing performance in a divided attention task, not considered by other works to the best of our knowledge, and in identifying strategic measures, which predict performance and describe the individual differences in decision making of subjects. The defined predictors can be generalized to some extent to divided attention tasks in which the individual simultaneous tasks are similar to each other, by requiring the same capabilities and types of user actions. Our efforts reinforce the importance of analyzing strategies in complex tasks.

## Methods

### Design of the DA Game

In this section we elaborate on the design process of the DA Game. First, we describe the graphic elements. Then the parameters are presented, which might influence the difficulty of the game, followed by the description of our choices for these variables.

**Fig 1:**
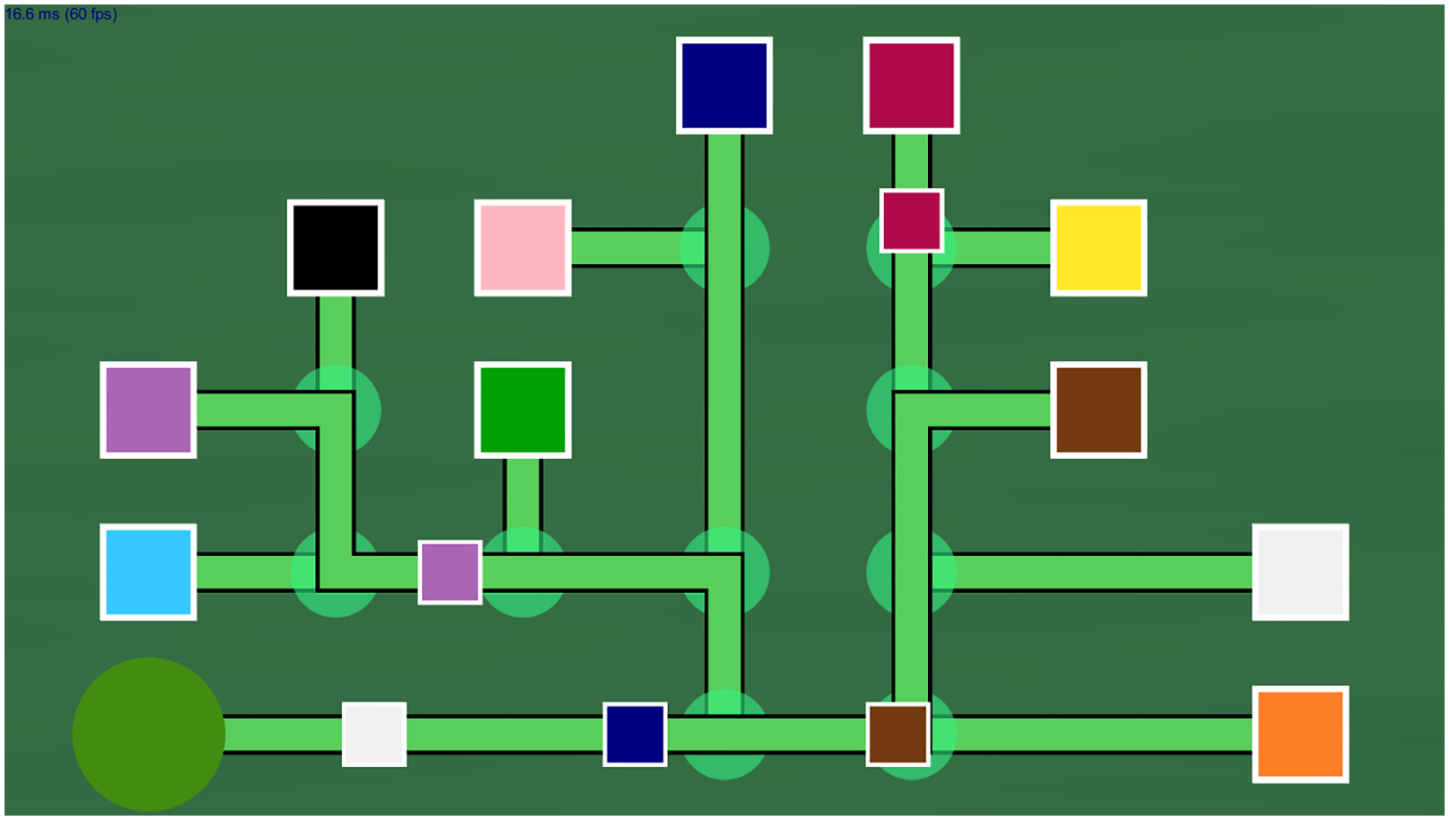
Snapshot of the Divided Attention Game. The small, colored squares emerge onto the tracks from the big green circle, are moving continuously along the green paths and can change direction at the switch nodes (the smaller transparent green circles). A single unambiguous path can be assigned to each small square, which has to reach its corresponding destination represented by a larger square of the same color. The player can flip the switches through mouse clicks; for example on this snapshot the switch in front of the small blue square should be switched so that the square turns left towards the blue destination. The switches on the paths of the brown and purple square are in the correct states at the moment.

The DA Game was implemented using Unity 3D. The graphic design was simplified compared to the Train of Thought from Lumosity. The game elements are represented by simple geometric shapes; a snapshot is shown on Fig 1. The moving units, i.e. trains, are represented by small squares; the destinations are denoted by larger squares respectively; the tunnel, from where the moving units emerge onto the tracks, is a big circle and the switches are displayed as smaller transparent green circles. The stationary elements of the track (tunnel, switches and destinations) are rendered onto a grid and connected by vertical and horizontal green paths, representing the rails. The colors of the destinations are monochromatic and were chosen to be easily distinguishable. The moving units are entering the tracks one by one, are moving continuously on the paths, can make 90 degree turns at the switches and the player has to guide them to their color-matching destinations. The switches can be flipped through mouse clicks in order to change the direction of the tracks and modify the path of the moving units. Each moving unit represents a distinct task in the game, a single unambiguous correct path can be assigned to it, and may require multiple user actions which may be performed in an arbitrary order. The terms *task* and moving unit will be used interchangeably in the rest of the paper.

#### Difficulty variables

The Train of Thought game is organized into different levels determined by the number of distinct destinations in one gameplay. This is the most evident way for representing the difficulty level. However, the difficulty depends on several other aspects as well: binary tree structure, layout of track elements, reflected version of layout, task sequence, distance between consecutive tasks within the sequence and speed of the moving units. These general difficulty parameters are described in detail next, and then we specify our choices.

**Fig 2:**
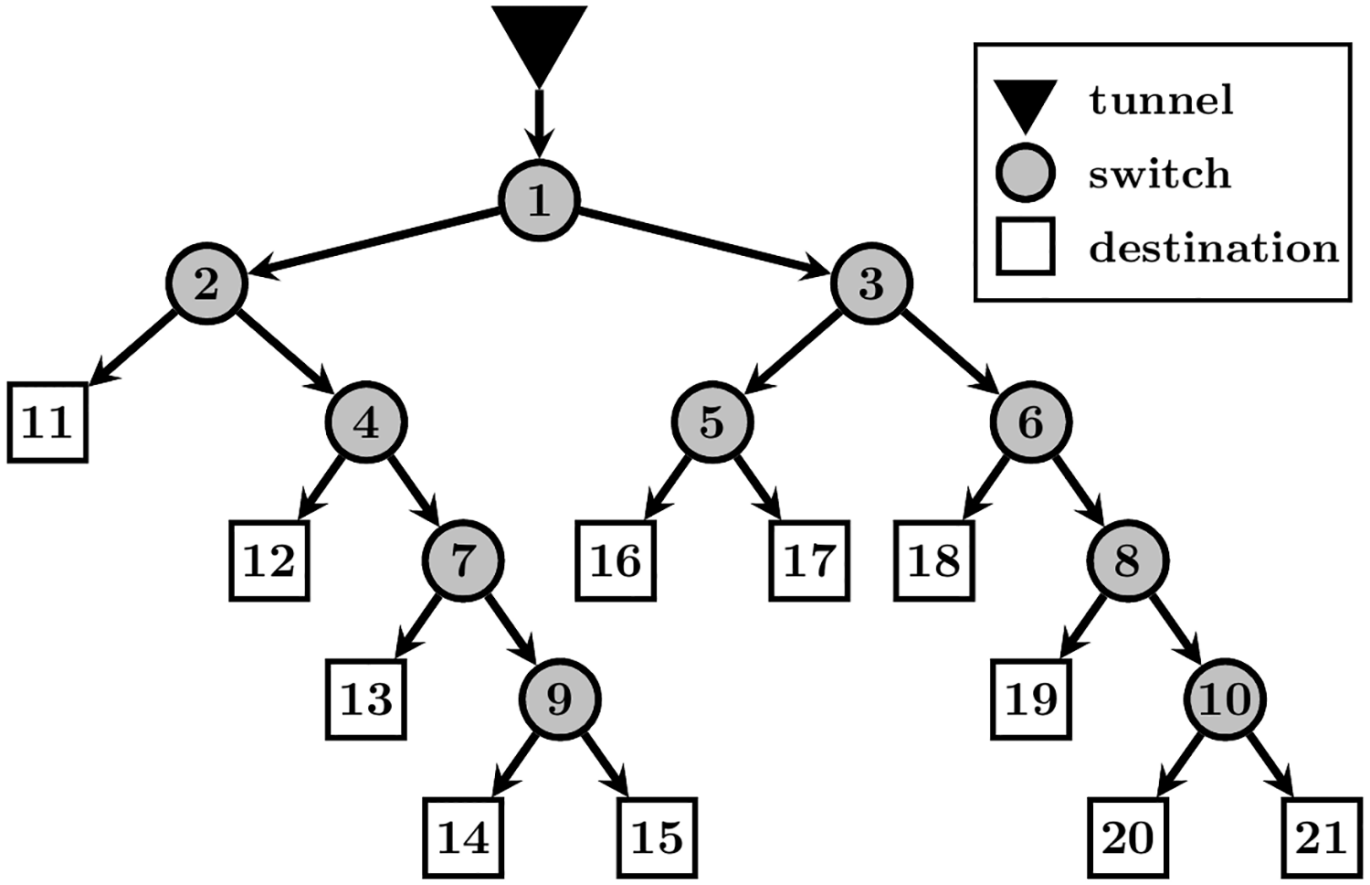
Example of binary tree representing the structure of the stationary track elements in the divided attention game. The root node represents the tunnel from where the moving units emerge, the internal nodes are the switches, the leaves are the destinations and edges are paths between the track elements.

The structure of the stationary track elements can be represented by a *binary tree*, where the root node is the tunnel, the internal nodes are the switches, the leaves are the destinations and the edges are the paths between the elements. An example tree with 11 leaves is shown on Fig 2. Increasing the number of leaves results in increasing the number of possible distinct tasks in one gameplay, meaning that a more complex mental structure has to be managed by the player.

The term *layout* refers to the collection of the 2D grid coordinates of the track elements. The coordinates from one layout can be *geometrically reflected* giving rise to different versions of the same layout. The *task sequence* is the succession of the moving units in one gameplay. The number of distinct tasks is determined by the number of the leaf nodes in the binary tree, since the id number of the leaf node determines the route a moving unit should follow (for example, referring to the tree from Fig 2, the correct route of a task with number 20 is 1–3–6–8–10–20). Accordingly, although the user might have a different order of actions, two tasks are considered the same, if their destinations match.

The *distance between consecutive tasks* within a sequence and the *speed of the moving units* are two other factors, which influence difficulty: the closer two tasks are in space and time or the higher the moving speed of a task is, the more efforts are required by the player to maximize performance.

Below we list our choices for the difficulty parameters in the DA Game.

- Binary tree structure: for the purpose of our experiments we have selected a single tree representation with 11 leaves (the one shown on Fig 2). Specifically, we always have 11 destinations which, together with the switches and tunnel, can be organized into the same mental structure.
- Layout: we have hand-designed 10 different layouts, which correspond to the binary tree structure from Fig 2, such that the tunnel (root node) is in the lower-left corner of the grid coordinate system.
- Reflected version of layout: there are four possible reflected versions of the same layout such that the distance ratios between the elements are preserved.
- Task sequence: in our case each gameplay contains a sequence of 42 tasks. Since there are 11 distinct destinations, tasks are repeated within the sequence. We have hand-designed five similar task sequences.
- The distance between two consecutive tasks: this was set to a constant value, corresponding to 3.96 seconds at the default speed.
- Moving speed: the default speed in the DA Game is 100 and corresponds to 8.8 seconds for a moving unit to get from the tunnel to the first switch node on each of our 10 layouts.

The selection of the 42 tasks contained in a sequence, the distance between consecutive tasks and the default speed is based on empirical considerations after conducting pilot studies. The other three parameters (layout, reflected version of the layout, and task sequence) can be combined to obtain a total of 200 (= 10 layouts x 4 reflections x 5 task sequences) different gameplays. Furthermore, the colors of the 11 destinations are randomly distributed before each gameplay, and accordingly this also determines the colors of the moving units within a task sequence. The reason of our choices is to have gameplays of similar complexities while minimizing the occurrences of visually similar temporal sequences.

### Participants

We have performed a series of experiments with 10 participants, with four females and six males, aged between 25 and 30 (mean age 27 years, SD = 1.76). The volunteers had normal or corrected-to-normal vision and reported no attention disorders nor color vision deficiency. Also they were naive in the sense that this was the first time they encountered the DA Game as well as did not have prior experience in Lumosity games. No other pretests were completed by the subjects, and they were instructed that data about their gameplays will be logged for further analysis. The participants were asked to sign a consent form before the experiments, and the study was approved by the Ethics Committee of the Faculty of Informatics, Eötvös Loránd University.

### Experimental procedure

We assessed the divided attention and working memory of 10 volunteers. The subjects were asked to play with the DA Game in identical conditions in terms of the environment and equipment. Their performance was monitored and all the game events were automatically logged for later analysis (e.g., mouse clicked, destination reached, etc.).

A recording environment was set up for the experiments and each test was run under supervisory guidance. The subject was sitting at a distance of approximately 60 cm from the computer screen. Similar light conditions were assured, background noise and other potential interferences were minimized. The same computer and mouse were used to play the game during the whole experiment. Subjects were not permitted to share their results with their peers, and adjusting the mouse sensitivity was also not allowed. The DA Game was displayed in full screen mode on a 23 inch full HD monitor and the sampling frequency was of 60 Hz.

A total of 10 sessions were conducted for every participant across 2-3 weeks, each session spanning about 30 minutes and consisting of 6 gameplays (called *trials* in the following). As a reference point, we note that a gameplay lasts about 3 minutes at the default speed of 100. In most cases every session was scheduled on a different day, or at least a few hours break was required between the sessions. After sessions we had brief interviews with the subjects about their experiences and took notes. We restricted the number of trials per session, in order to avoid the effect of fatigue from sustained attention and focused mouse movements. Furthermore, participants were given a choice to take self-timed breaks at the end of each trial within sessions.

The score of the player in one gameplay is given by the number of successfully completed tasks, i.e. the number of moving units out of 42 that reached their correct destination. This served as the measure of user performance. Also, in our experiments every time a possibility of the 200 possible layout, reflection and task sequence combination was automatically selected with equal probability.

#### Difficulty manipulation and experimental phases

The variable that determines the level of difficulty in the DA Game is the speed of the moving units, referred to in the following as the *game speed*. This was changed according to fixed rules.

In the first trial of every participant, the speed was set to the default value of 100. Before each subsequent trial, the game speed was adjusted by integer values, corresponding to percentages, according to the score of the preceding trial. If the player achieved a favorable score, then in the next trial the speed was increased, while a low score implied a decrease of the speed. The amount of speed change is determined by three different predefined sets of rules, summarized in Table 1. Based on these policies, the 10 sessions are split into three separate phases:

- Phase 1: beginner level. It consisted of the first 4 sessions (24 trials) and the speed was changed only by a maximum of 1% between trials;
- Phase 2: intermediate level. It contained sessions 5 and 6 (12 trials). Conditions for increasing the speed were less stringent;
- Phase 3: challenging level. It covered the last 4 sessions (24 trials) and the game speed could change even by 10% between trials.

**Table 1:** Rules for changing speed of the game in experimental phases, based on the score from the previous trial.

Score		42	41	40	39	38	37	36	35	34	33	32	31	30	29	28	27	26	<25
Speed change (%)	Phase 1	1	0	0	0	-1	-1	-1	-1	-1	-1	-1	-1	-1	-1	-1	-1	-1	-1
	Phase 2	6	4	2	0	0	0	0	-1	-2	-3	-4	-5	-6	-7	-8	-9	-10	-10
	Phase 3	10	10	10	10	8	8	8	0	0	0	0	0	0	-2	-4	-6	-8	-10

### Strategic predictors of performance

We carried out an extensive analysis of the experimental data by carefully considering a number variables, defined based on the characteristics of the actual game situation and the types of user actions, consisting of mouse clicks. The conclusive result is the identification of four strategic predictors, which characterize the performance of the participants. For better understanding, we first differentiate two types of game situations through an illustrative example. Then, we provide the definition of the four measures referring back to this example.

#### An illustrative example

**Fig 3:**
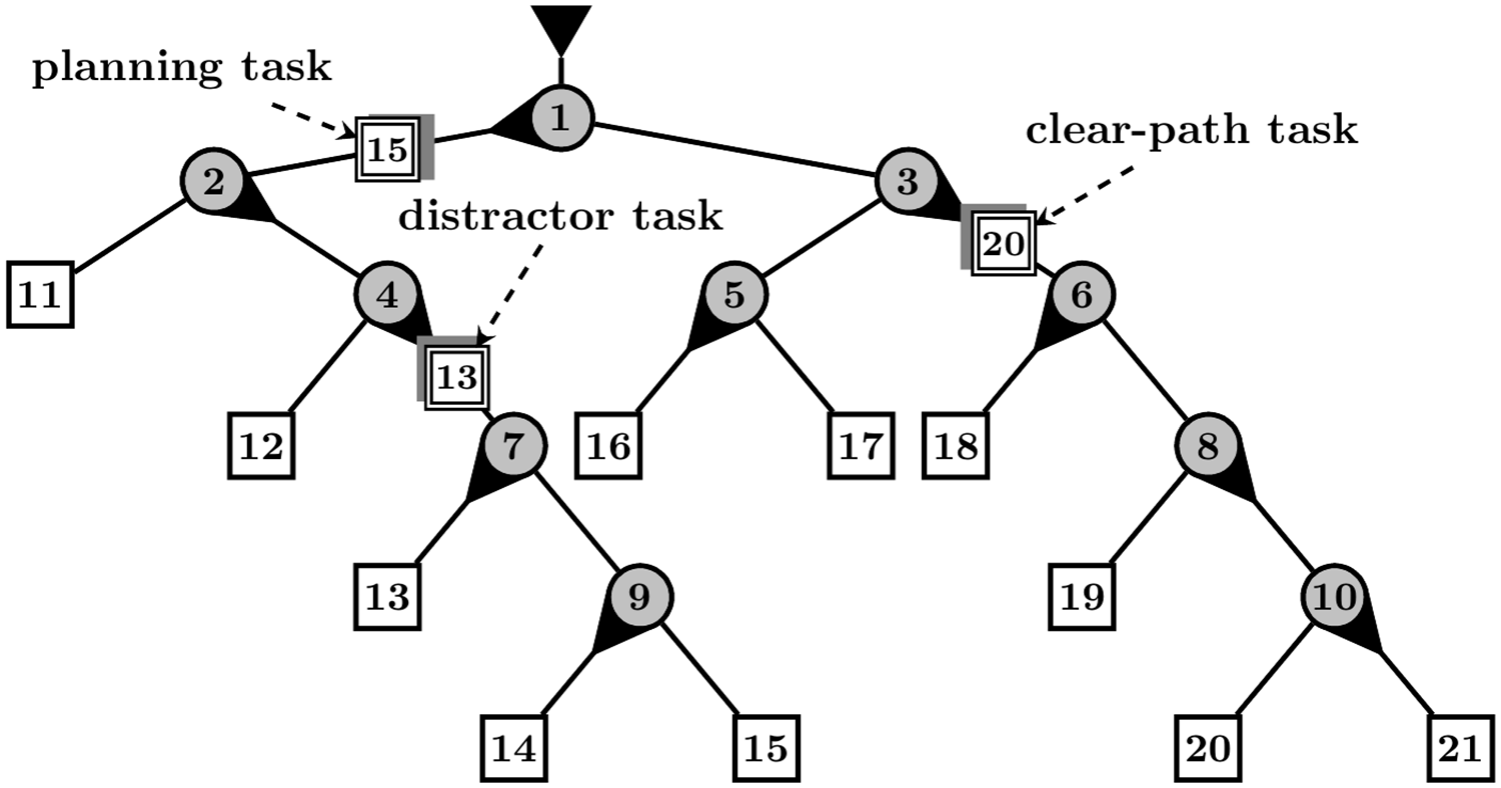
Illustration of the planning and clear-path (non-planning) game situations, and the four types of strategic decisions. The moving unit #15, having a distractor (#13) on its future path, is called a planning task; #20 is a clear-path task since its future route to be followed is clear from other tasks. The strategic decisions defined in the paper (and examples on the present illustration) are: (i) planning switch flip (flipping switch #9 for task #15), (ii) primary task selection (flipping switch #6 or #10 for task #20), (iii) within task switch flip (#6 followed immediately by #10, or in reverse order) and (iv) primary action selection (# 6 for task #20).

Let us consider the following example depicted on Fig 3. Three simultaneous tasks can be seen on the figure, heading to destinations no. 13, 15, 20, and the directions of the switches are also depicted. In the remaining of this section we will refer to these examples in parentheses. The user may have tasks, which—according to the states of the switches—require no action (task #13) and others that do (tasks #15 and #20). The action of switching may not be possible at the moment due to other ongoing tasks (flipping switch #7 for task #15 has to be postponed because of #13). On the other hand, if switching is possible, we differentiate two types of tasks:

- switching can be performed with no other ongoing task that would constrain the time of the action (flipping switch #6 or #10 for task #20),
- switching can be delayed with another ongoing task that could constrain the time of the action (flipping switch #9 for task #15, although task #13 is still on the future path of #15).

Accordingly, two types of game situations can be differentiated when a switch is possible: either the path is clear from other moving units—this is a *clear-path* task situation, or the path is not clear yet—this will be called a *planning task* situation. If the path is not clear yet, then the unit ‘in the way’ can be seen as a distraction, or obstacle, or action delay constraint. Note that if switching could be delayed for a task, but no distractor is present, then this is not seen as a planning task (flipping switch #10 for task #20 is not urgent but possible).

#### Definition of the strategic decisions

The *planning ahead* or simply *planning* action of the user is defined as flipping a switch for a moving unit that has a distractor task on its future path (switch #9 for task #15). The action is called a planning switch flip. Alternatively, the player might also choose to wait until the distractor moving unit is no longer on the path of the planning task and click the switch then. However, this leads to a clear-path situation. In turn, a planning interval ends not just as the user performs a planning switch but also when the distractor moving unit is no longer on the path of the task.

Because in the DA Game switch flips are often possible on the paths of multiple moving units simultaneously, the player has to decide how to control the order of the tasks. This is referred to as a task coordination problem. The primary task is the moving unit closest to the first switch on its path, where a switch flip is needed and possible. We are interested whether the player selects the primary task, or another secondary moving unit. Accordingly, *primary task selection* is defined as flipping a switch on the path of the primary task (task #20 has switch #6 the closest on its path; flipping switch #10 also counts since it is on the path of task #20).

In addition, more than one switch might need to be flipped on the path of the same task at a given time (switches #6 and #10 for task #20). Therefore, we define the *within-task switch flip* as the case when two consecutive responses are given within the same task.

In close relation to task coordination, we also define the action coordination problem. Out of all the switches where mouse click is required and possible, the primary switch is the node where a moving unit arrives the earliest (switch #6 is the closest on the path of task #20). *Primary action selection* means flipping the primary switch.

To summarize, the four strategic decisions of the players are: planning switch flip, primary task selection, within-task switch flip, and primary action selection. In order to compare individual differences between players, these strategy measures will be analyzed in terms of proportions:

- proportion of planning: we counted those tasks, in which a planning switch was performed and divided it by the total number of cases, when planning was possible;
- proportion of primary task selection: we counted the number of switch flips for the primary task and divided it by the total number of switch flips (for both the numerator and denominator only those cases were taken into account when multiple tasks were present to choose from at the moment of the click);
- proportion of within-task switch: the number of cases when two consecutive switch flips were made for the same task was divided by the total number of switch flips, which could be followed by a response within the same task at the moment of the switch;
- proportion of primary action selection: this was defined analogously to the proportion of primary task selection. We took into account only those cases when multiple switch flips were possible at the moment of the click.

A few notes have to be made. First, a switch flip may simultaneously count as any two of the strategic decisions defined. In such overlapping cases we do not accord priority to either, but both definitions are taken as valid. Second, the primary switch always belongs to the primary task, however, the inverse is not true in all cases (for example, on Fig 3 task #20 is the primary task and switch #6 is the primary switch, but flipping switch #10 also counts as the selection of the primary task). Third, in the computation of the proportions, moving units, which were already on the wrong path were neglected in all cases; and also erroneous switch flips and corrective switch flips were excluded. For the sake of clarity, we provide below the definition of these latter two types of actions.

The mouse clicks of the players can be divided into two main categories: correct or erroneous. In the analysis of the strategic predictors we were only concerned with the correct switch flips, which can be further partitioned into planning, clear-path (or non-planning) and corrective switch flips. The first two types of switches were defined at the beginning of this section. The corrective responses are corrections of preceding erroneous switch flips, when the player immediately noticed the mistake committed and clicked again the same switch.

In order to analyze the performance of the participants from the experiments and its relation to the four strategic measures identified in this work, we exploited the following statistical methods. To compare the scores between participants across phases, separate one-way ANOVA models were applied. To assess whether the identified strategic measures would predict DA Game performance, regression analysis was carried out. Specifically, in order to account for the within-subject correlations of the repeated observations, generalized estimating equations [50, 51] were used with the identity link function and the parameter estimates are reported for the case of exchangeable working correlation matrix. We note that switching to first-order autoregressive correlation structure also gives similar estimates in all cases, thus not contradicting our conclusions. To compare GEE models, the marginal *R^2^* [52] and the quasi-likelihood under the independence model criterion (QIC) [53] measures are reported. Also, to assess the association between the strategic measures and score, repeated measures correlation [54–56] coefficients are calculated.

## Results

### Performance of participants

**Fig 4:**
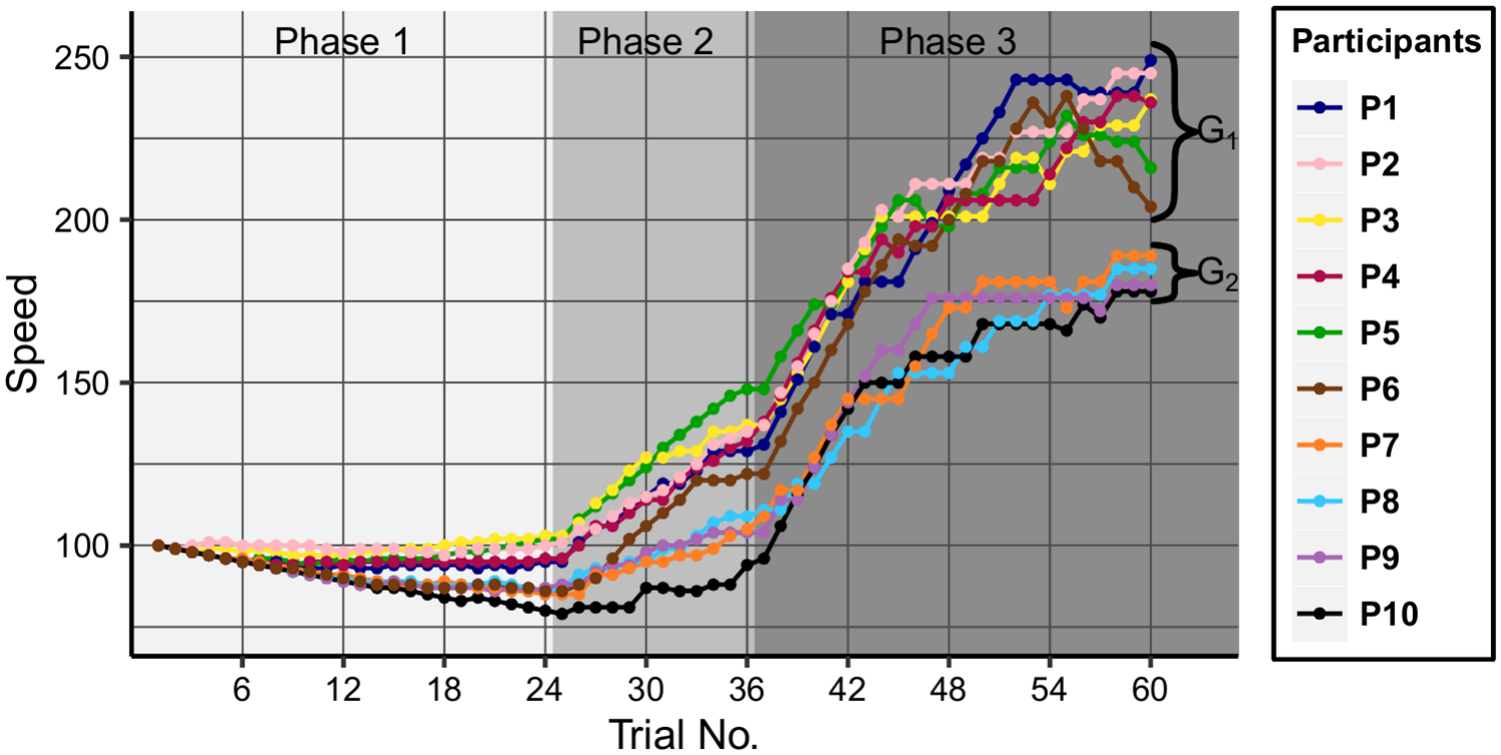
Participants’ performance. The game speed values reached (y-axis) are shown across the trials (x-axis) of the experiment.

Fig 4 illustrates the performance of the participants, by depicting the speed values and also highlighting the three phases of the experiments. Two groups are formed: the first group (G_1_) consists of 6 participants (P_1_–P_6_) who have their last speed value greater than 200, while the second group (G_2_) is formed of the remaining 4 participants (P_7_–P_10_) having the last speed values below 200. The subjects in our analysis were ordered by their last speed values. The two groups are formed very early, in the second part of Phase 1 and remarkably P_6_ belongs to G_2_ in Phase 1, then in Phase 2 moves closer to the members of G_1_.

The speed progress curves of the members of G_2_ are nearly flat after the 47^th^ trial, meaning that these participants could not progress significantly in this part of the experiments. In the same time, variance in performance of G_1_ members increased, with values still remaining above the threshold of 200 speed units.

The game speed was changed based on the score of the participants, applying fixed rules. The score of the first trial was typically very low for every participant, so it was excluded from further investigations. To analyze the performance differences between participants, separate one-way ANOVAs were used per experiment phase with the score per trial as dependent variable. The results are displayed in Table 2. Statistically significant differences were found between the means of scores from the first phase of the experiments, *F*(9, 220) = 8.48, *p*<0.001. A post hoc Duncan analysis reflected the two performance groups identified in our experiments, as shown in Table 2. P_10_ had the lowest mean score which was significantly different from all other values. The mean score for P_6_ was situated between the two performance groups, while all members of G_2_ were significantly different from the remaining members of G_1_. On the other hand, the one-way ANOVA indicated no statistically significant differences between the means of the participants’ scores for Phase 2, *F*(9, 110) = 1.47, *p* = 0.17, and Phase 3, *F*(9, 230) = 0.69, *p* = 0.72.

**Table 2:** Score means and standard deviations and comparison of participants’ performance in experimental phases.

		Phase 1	Phase 2	Phase 3
	P_1_	39.78 (1.83)^a,b^	40.17 (1.64)	35.96 (3.92)
	P_2_	40.61 (1.16)^a^	40.42 (1.00)	35.67 (3.92)
	P_3_	40.65 (1.58)^a^	39.92 (2.02)	35.54 (3.81)
	P_4_	39.96 (1.74)^a,b^	40.67 (1.23)	35.04 (3.58)
	P_5_	40.35 (2.25)^a^	40.67 (1.56)	34.08 (4.30)
	P_6_	38.70 (2.36)^b,c^	40.33 (1.44)	34.75 (5.69)
	P_7_	37.96 (2.79)^c^	39.75 (1.29)	34.96 (3.78)
	P_8_	38.09 (3.29)^c^	39.50 (1.68)	34.58 (2.21)
	P_9_	37.83 (4.12)^c^	39.42 (1.16)	33.96 (3.64)
	P_10_	35.87 (3.11)^d^	39.17 (1.90)	34.46 (3.74)
**ANOVA results**	df	(9, 220)	(9, 110)	(9, 230)
	*F* value	8.45	1.47	0.69
	Pr>*F*	<0.001	0.17	0.72

Standard deviations are shown in parentheses. Letter superscripts refer to the groups produced by the Duncan post hoc test.

**Fig 5:**
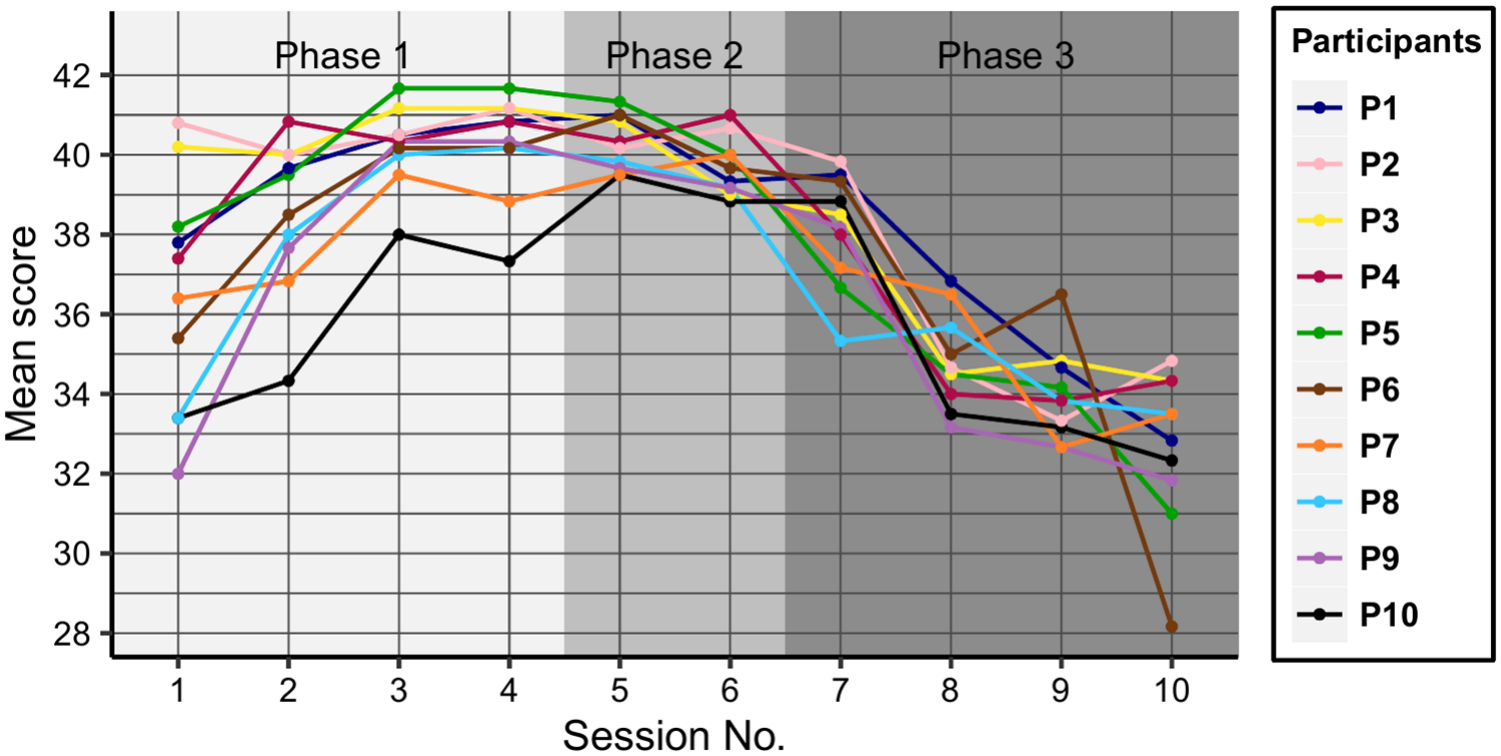
Mean scores across sessions.

The mean score has the maximum value of 42. Mean scores per sessions are meaningful in the sense that they reflect how the participants’ performance increases with practice and decreases with enhanced difficulty, as seen on Fig 5. The averages show an increasing pattern in Phase 1, since in this period the players are getting familiar with the game and the score is low at first but it increases with experience. In Phase 2 the values are high. During Phase 3 a decreasing pattern sets in, because, despite a lower score, the game speed was still raised according to the rules of this phase, thus resulting in players producing more errors.

### Composition of correct switch flips

**Fig 6:**
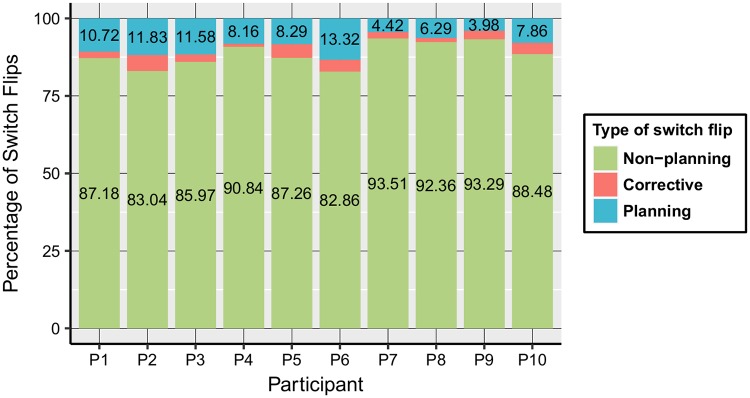
Overall composition of correct switch flips for participants.

We separated three types of correct switch flips: non-planning, corrective and planning ones. Fig 6 shows the percentage of each of the categories, separately for every participant. Most of the correct switch flips are non-planning (over 82% in all cases) and the percentages classified as corrective switches are small. The percentage of the planning switch flips lies between 3.98% and 13.32%, and the value of about 8% separates the two performance groups, with members of G_1_ above, and members of G_2_ below this threshold.

The percentage of the planning switch flips compared to the total number of correct switch flips already indicates the separation of the performance groups, to some extent. The goal of the upcoming analysis is to explain in detail the groups separated based on their performance, beyond validating the individual differences. It is shown that the identified strategic measures justify the achievement of the subjects.

### Analysis of the strategic predictors

**Fig 7:**
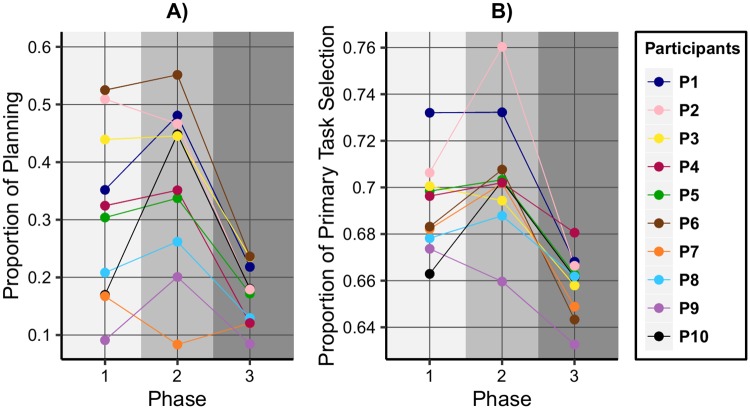
Strategic predictors across experiment phases, showing the differences between the performance groups.

Fig 7A depicts the proportion of planning switch flips, as defined previously, separately for every participant in each phase of the experiments. In order to provide a closer insight into the planning situations, it must be noted that the average number of tasks in the case of which a planning switch is possible is about 18. The possible planning situations might be overlapping, and often their duration might be too short (below 1 sec) for a mouse click, because the player needs to flip non-planning switches as well. However, sometimes the user ends the planning situation, by very quickly foreseeing it and preparing beforehand to flip the planning switch.

As illustrated on Fig 7A, in Phase 1 the performance groups are well-separated by a noticeable gap. We can draw a horizontal line at the hypothetical threshold of 0.25, which results in members of G_1_ being above and members of G_2_ below the line. In Phase 2 the planning proportion of two members of G_2_ remains below this splitting value, for one member it is slightly above, while in the case of P_10_, the proportion is increased dramatically up to almost 0.5. Remarkably, the planning ability of one person from G_1_, namely P_7_, decreases below 0.1, meaning that this person plans in less than 10% of the different situations. In Phase 3 all values are below the threshold, most likely because the players don’t have enough time to plan ahead. The planning proportions are decreased considerably, except for one participant (P_7_), in the case of whom it is slightly increased, but still remaining below the corresponding value from Phase 1.

Fig 7B shows the proportion of primary task selection per phases. The values reflect the two performance groups in Phase 1, with a gap between members of G_1_ and G_2_. Again we can notice a separating threshold of about 0.69, with members of G_1_ being above this value, with one exception: the value for P_6_, the subject whose performance moved between the groups, is now below the threshold. In Phase 2 the proportions increase, except for one participant (P_9_), for whom the distance from the threshold declined further. Because of the high game speed in Phase 3, it becomes difficult to select the primary moving unit and similar to the analysis of the planning proportions, all the values drop below the threshold.

The two features presented above highlight the differences between the groups separated according to the performances: in Phase 1 they were separated by a hypothetical threshold. We have also seen how the values for all subjects drop below this threshold in Phase 3.

In the analysis of the planning proportions, we observed that the value for P_10_ in Phase 2 was among the values of those from G_1_. Therefore, the next strategic measure, beyond further characterizing the subjects, provides an explanation for P_10_ being an outlier in this context.

**Fig 8:**
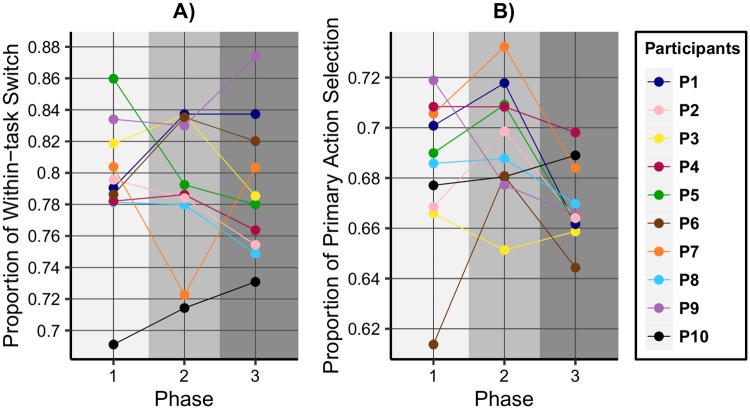
Strategic predictors across experiment phases, showing the individual deviations of participants from the performance groups.

Fig 8A shows the proportion of the within-task switch flips per phases. In Phase 1 the value of about 0.69 for P_10_ is far below the others, with the next value in this phase slightly above 0.78. In Phase 2 the proportion is marginally increased, but still remaining at the bottom at a considerable distance from the other subjects’ values, except from the value of P_7_. For this participant the proportion was decreased (remember that the planning proportion was also the lowest in Phase 2 for P_7_).

Fig 8B shows the proportion of primary action switch flips per phases. P_6_ had the highest proportion of planning in Phase 1, but the primary action selection proportion of about 61% is at a considerable distance from the next value in order. However, in Phase 2 the proportion is increased noticeably, while in Phase 3 it falls back again to the last place. This is consistent with the performance, which is decreasing at the end of the experiments at high speed values, as seen on Fig 4.

To assess whether the identified strategic measures would predict DA Game performance, two GEE models were used in a stepwise fashion. For this the proportions of the strategic predictors and the average of the scores were computed over sessions, separately for every participant. The repeated measures correlation coefficients presented in Table 3 indicate that performance was significantly and positively correlated with all four strategic predictors, with planning and primary task selection being strongly correlated with score (*r_rm_* = 0.75, *p*<0.001 and *r_rm_* = 0.65, *p*<0.001, respectively), and within-task switch and primary action selection moderately related (*r_rm_* = 0.30, *p*<0.01 and *r_rm_* = 0.43, *p*<0.001, respectively). There was a significant positive relationship between planning and primary task selection (*r_rm_* = 0.57, *p*<0.001), and a significant strong association between primary task and primary action selection (*r_rm_* = 0.71, *p*<0.001). Furthermore, planning positively and moderately correlated with within-task switch flip (*r_rm_* = 0.42, *p*<0.001).

**Table 3:** Repeated measures correlations between strategic predictors and score computed over sessions, separately for every participant.

	1.	2.	3.	4.
1. Planning switch flip				
2. Primary task selection	0.57***			
3. Within-task switch flip	0.42***	0.03		
4. Primary action selection	0.07	0.71***	−0.10	
5. Score	0.75***	0.65***	0.30**	0.43***

*N* = 100,

* *p* < 0.05,

** *p* < 0.01,

*** *p* < 0.001

In the first GEE analysis (Model 1), the within-task switch, primary task selection and primary action selection measures were entered together in the first step and planning in the second step. In the second model the order of the steps was reversed. The results are displayed in Table 4. Primary task selection and within task switch were significant predictors in Model 1 and together with primary action selection accounted for 51% of the variance.

**Table 4:** Results of generalized estimating equation (GEE) analysis with strategic measures predicting DA Game performance.

	Model 1	Model 2	Full Model
**Model 1**			
Primary task selection	55.13***		−7.73
Within-task switch flip	11.55***		7.75**
Primary action selection	−3.07		51.50***
**Model 2**			
Planning switch flip		15.82***	15.12***
$R_{marg}^{2}$	0.51	0.47	0.71
$\Delta R_{marg}^{2}$	0.19	0.24	
QIC	477.45	529.91	290.63

*N* = 100,

* *p* < 0.05,

** *p* < 0.01,

*** *p* < 0.001.

GEE is used with the identity link function. Unstandardized regression coefficients are shown for the case of exchangeable correlation structure. Full model refers to regression with all four strategic measures as predictors. The model selection measures are the marginal *R^2^* and the quasi-likelihood under the independence model criterion (QIC).

In Model 2, with planning as the single significant predictor in the first step, the amount of explained variance was 47%. Planning accounted for an additional 19% of the variance when it was added to Model 1, and while primary action selection became a significant predictor in the full model, primary task selection was no longer significant. Adding the other three predictors to the second regression model also resulted in significant incremental validity over and above planning (24% additional variance). To sum up, the identified strategic measures in the full model explain a large proportion of variance in DA Game scores (71%), with three variables being significant predictors. Inspecting the criteria measure for GEE, the best fitting model to the data was the full model having the smallest QIC value.

## Discussion

### Summary of results

In this study we identified four strategic measures, which justify the achievement of the participants in a divided attention task. The strategic decisions presented were labelled as planning switch flip, primary task selection, within-task switch flip and primary action selection. They were analyzed in terms of proportions in order to provide a proper comparison of time intervals and individuals, by taking into account the number of possibilities present for making the particular decisions.

The presented results indicate that two of the strategic predictors refer to the differences between the observed performance groups (see Fig 7), while the other two highlight the individual deviations from these groups (see Fig 8). In the case of proportion of planning and of primary task selection, we have seen how a hypothetical threshold separated the performance groups in Phase 1 of the experiments, and how the values drop below the threshold in Phase 3. The proportion of within-task switch flips indicated that, although P_10_ started to plan ahead significantly better than the rest from G_2_, the performance did not improve considerably because this strategic measure still remained low. The proportion of primary action selection showed that, although P_6_ had the highest proportion of planning along the experiments, in Phase 1 this subject’s performance was similar to those of the members from G_2_, because of a considerably low proportion of primary action selection.

Figs 7 and 8 depict the proportions computed over the experimental phases for highlighting the individual differences in strategic decisions between subjects. In order to examine whether the strategic measures predict performance in a regression analysis, we computed the proportions over sessions. In order to account for the correlation between the scores of the same subject across sessions, a GEE analysis was used. A repeated measures correlation analysis revealed that all four strategic measures were significantly and positively related to performance (see Table 3).

The planning strategic measure had the highest correlation with score. It took an extra amount of effort to identify this variable, than the other three more straightforward predictors. Accordingly, stepwise GEE models were applied, where planning was entered in separate steps. Although, the number of the planning switch flips was very low when compared to non-planning switches (see Fig 6), the variable defined as the proportion of the planning switch flips to the number of tasks which included a possible planning situation, was found to be the most important predictor of the players performance in the GEE analysis.

Table 4 summarizes the results of the GEE analysis. In Model 2, as the single predictor variable in the first step, planning accounted for a considerable amount of variance (47%), almost as much as the other three strategic measures together (51%) in the first step of Model 1. When planning was added to Model 1, it accounted for an additional 19% of variance in performance above and beyond the other variables. This value is slightly less, than the additional variance the group of three could account for when the order of the steps was reversed (24%). Finally, three variables of the four (planning, primary action selection and within-task switch flip) were found to be significant predictors of score in the full GEE model, in which the strategic measures accounted for over 70% of the variation.

### Connection between the four strategic measures and relation to other strategies

In the Methods section we noted that strategic decisions can be overlapping sometimes, i.e. a user action is counted simultaneously as two or more of the four cases. Particularly, primary task and action selection are in close relation, since the primary action always belongs to the primary task. Their tight connection is underlined by our evaluations too. On one hand, their significant positive correlation was the highest (0.71) among the correlations between the strategic measures (see Table 3). On the other hand, in the GEE analysis conducted, only primary task selection was a significant predictor of performance out of the two in the first step of Model 1 (see Table 4). However, their significance was reversed in the full model, most likely because planning also referred to the performance group differences and highly correlated with primary task selection, while there was no correlation between planning and primary action selection.

Despite the near zero correlation, the within-task switch flip strategic measure is closely related to primary task and action selections. In particular, it might represent an interfering decision of the user, when multiple tasks and actions are possible. In such cases the user has to decide whether to continue with an action within the same task, or select the more urgent one. In any case, the within-task switch flip was a significant score predictor in the GEE analysis conducted.

Besides the four strategic decisions described in the paper we have considered several other variables such as the types of user errors (omission and commission) and their ratio compared to the overall number of errors; time/distance remaining until last possibility of performing an action; the number and ratio of corrective switch flips; the number of cases when no correct switch flips were possible in the middle of gameplay but players still make an action; features computed from mouse movements, among others. We analyzed these variables but none of them reflected the performance groups seen in our experiments and did not improve the fit of the regression models. Consequently, this paper focuses on the description of the four strategic decision parameters, which had significant correlations with performance.

The identified strategic measures in this work can be associated with strategies investigated by others as well. Planning might be related to the holistic planned strategy of Peña et al. [31], which led to better performance than the other two response patterns identified in their study, and is characterized by an active integration of task elements and initial planning of the responses before acting. Similarly, the visual scanning strategy identified by Kallai et al. [49] also involves active visual exploration of the environment and was shown to be strongly related to more accurate performance. The planning switch flips in the DA Game are actions performed before they would become critical and assume processing of the relations between the elements of the environment. Subjects who plan ahead to a greater extent, possibly possess a better mental representation of the game structure and recognize available hidden actions more often.

Primary task and action selection is similar to the well documented optimal strategies in Space Fortress, which consist of prioritizing some actions of the player over other ones (see, e.g., [25] and the references therein). Task priorities have been shown to influence performance in a different, dual-tasking setting too [42], where a visual-manual task, executed with secondary priority, yielded high dual-task interference with an auditory-vocal task. Wang, Proctor and Pick [33] showed that participants in their experiment with a synthetic work environment favored a task out of four different tasks more when its payoff was high then when it was low, providing evidence that strategies reflect the relative importance of the tasks. The adaptation of strategy was observed by Janssen and Brumby [44] as well in a driving-while-dialing scenario, where participants gave greater priority to one task over the other when instructed so. Task organization and prioritization in general may be critical and have serious negative consequences [30].

The within-task switch flip strategic measure is essentially the same as the within-task response probabilities of Hambrick et al. [24], with the note that the parallel tasks were different from each other in their study.

### Notes on ability and personality variables

Performance in the DA Game might also depend on special individual abilities of the players. Task specific skills can have a positive impact [43], for example, the handling of the computer mouse in our case. Better attentional focus should lead to higher performance [13, 25, 27]. Higher working-memory capacity presumably would be associated with better performance, because it can help to switch between tasks, by storing information related to a task, while simultaneously performing another [13, 20, 24, 30, 32, 46]. It was found to be a strong predictor of multitasking performance in several studies [13, 21, 24, 27, 32]. We hypothesize that the planning strategic measure is associated with higher working memory capacity, since it has an effect on reducing near future cognitive load and it was shown to have crucial importance in achieving better performance in the DA Game.

Primary task and action selection refer to a spatial ability of determining the shortest distance between objects out of multiple choices available. Although not choosing always the primary task or action does not necessarily degrade performance, these are the ones that should be handled or carried out the soonest in order to minimize the immediate number of errors in the DA Game.

The within-task switch flip variable is connected to self-interruptions, which have been proven to affect performance in different multitasking settings (e.g., [45, 46, 57]). In our case, the lower the proportion of within-task responses, the higher the self-interruption rate. Further analysis is needed to see, which is the correct decision in the DA Game; namely responding within the same task, or interrupting the control of the ongoing task and selecting the more critical response. This question is, however, complex, since it might depend on the abilities of the player, on the game situation and also on the speed of the game. Our results indicate via the correlation and regression analyses that selecting the primary action seems more important.

An individual’s emotional experience might be an important predictor of performance, too [30, 34]. For instance anxiety is expected to have a negative impact on performance [20]. Although we did not measure anxiety explicitly, based on the subjective reports of the participants, we can state that they were more nervous during Phase 3 of the experiments, due to the high game speed values. Accordingly, more errors were committed in this part as shown on Fig 5. A related personality trait is neuroticism, which had a significant negative relation with performance in other studies [20, 34]. Negative feelings in general can impair performance [22].

Further studies should investigate the above mentioned research questions and assumptions related to ability and non-ability factors, possibly with a larger sample of participants. The sample in this study was relatively small, which limits our conclusions. Another potential pitfall would be the absence of motivation of participants. However, based on subjective reports after each session, we are confident that all participants were determined to reach the highest score possible.

Nonetheless, we suggest that even if such abilities could be controlled (or selected) one way or another, strategy would continue to remain a significant predictor of performance. This would be in congruence with other studies. For example Schunn and Reder [23] found that the correlation between strategy adaptivity and performance was not mediated through indirect correlations with seven cognitive abilities. Hambrick et al. [24] also showed that strategy use accounted for a large proportion of variance in multitasking, above and beyond ability factors.

### Contribution and limitations

Our work demonstrates that it is important to evaluate strategies when analyzing complex task performance. This is in line with other studies which, by contrast, all used different environments. Arthur et al. [25] conveyed to participants well documented optimal strategies of a complex task. Gonzalez [29] demonstrated that relying on simple heuristics led to poorer performance than acquiring more complex and context-based knowledge in a dynamic decision making task. Kallai et al. [49] showed that search strategies determined overall performance in a spatial orientation task. Performance was defined as the time needed for the subject to locate and move onto the target, and strategies were characterized as motion patterns in spatial navigation. In a similar study, Peña et al. [31] demonstrated that the different response patterns in a spatial dynamic task lead to large performance differences. Hambrick et al. [24] found that strategy accounted for a large proportion of variance in a synthetic work paradigm, above and beyond other variables. Hoffmann, von Helversen and Rieskamp [48] highlighted the importance of considering people’s cognitive strategies to understand performance in demanding environments. Duggan, Johnson and Sørli [45] highlighted the importance of interleaving strategies in the management of simple office-based tasks. Janssen and Brumby [43] provided a detailed analysis to prove that people adapt their interleaving strategies to meet specific criteria and to achieve near optimal performance.

Besides characterizing the strategic predictors, this study also showed that the performance of the participants improved with practice and decreased considerably with task difficulty (see Fig 5). We manipulated the pace of the divided attention game according to performance in order to keep players motivated and engaged, and to see the effects on problem solving in a divided attention task. The improvement can be seen in Phase 2, where the average scores across sessions were greater than 39, i.e. performance was above 90%. We mention that because the DA Game is a complex dynamic task, it is challenging to achieve perfect score even for experienced players. In Phase 3 we observed that the strategies of the subjects were adapted to the more demanding conditions. This is underlined by two strategy variables: the proportion of planning and of primary task selection in Phase 3 dropped under a hypothetical threshold, which separated the performance groups in Phase 1. It is appealing to hypothesize that drop in performance in Phase 3 is due to the fast pace of the game that increases the cognitive load, which induces players to switch to less demanding strategies (see [48] and the references therein) and which was also shown to decrease the visual field of an individual [58]. The difficulty here can be attributed to time constraints, which have been found to influence performance negatively. For example Gonzalez [29] found that participants under high time constraints performed worse than those under low time constraints in a dynamic decision making task, even after additional practice. Moon and Anderson [41] also found that time pressure impaired performance in a target interval estimation task. Task difficulty in general influences performance negatively (see, e.g., [43, 45, 57]).

Note that the average scores in the first session, shown on Fig 5, suggest a close to unimodal distribution. This remark is supported by the Shapiro-Wilk test as well, *W*(10) = 0.96, *p*>0.05. However, the values for members of the G_1_ performance group are above those of G_2_. In turn, our results indicate that distinct strategies influence general performance and give rise to different and diverging learning trajectories.

Our studies are limited to a specific task and a small number of subjects, which calls into question the generalizability of the results. At the same time, our restricted group of participants (normal subjects aged between 25-30, whose average scores in the first session suggest an unimodal distribution) produced significant separation in the progresses. Such separation would be flattened out for a larger variety of subjects. Another important point to consider is that we made efforts to reduce the complexity of the DA Game by restricting the variability of the difficulty parameters. This way we limited the number of possible outcomes. As a result, consecutive gameplays are of similar complexity, but still complicated enough so that the chances for remembering visually identical temporal sequences is minimal. Furthermore, the manipulation of the speed as the only difficulty parameter also contributes to the separation in progresses. These carefully crafted circumstances helped us to find and highlight relevant explanatory variables.

Despite the limited number of participants, our significance tests show that the performance measures identified and characterized are important cues that explain achievements of the participants. Furthermore, the individual differences in strategy measures cannot be attributed to chance variation, because they were strongly associated with varying performance. Recognizing strategies in general is a key to understanding how people solve problems [48].

We close this section by noting that it can be difficult to break away from well-practiced problem-solving routines. In turn, training which takes advantage of potential strategies used by other practitioners can help in improving individual performances.

## Conclusions

In this work we described a game-like divided attention task, which challenges our brain by presenting competing information and by requiring frequent shifts of attention during a several minute period. Players need to respond rapidly to changing visual information and are forced to avoid errors by focusing on the more important tasks first. A series of experiments were conducted and evaluated. We demonstrated that practice increases performance, task difficulty has an impact on the strategies employed, which in turn influence achievement. More importantly, we identified four strategic measures as a result of an extensive analysis by carefully considering the types of user actions in relation with the circumstances of the game situations. We showed that these predictors explain a large portion of variance in performance and highlight certain individual differences.

Two of the strategic decisions refer to prioritizing one task or one action over others, which could also be rewarding at the same time. One predictor refers to the choice of a response within the same task and is closely related to self-interruptions. The fourth measure of strategy is called planning and consists in thinking ahead, i.e. recognizing upcoming future actions and responding before they may become critical. This has the effect of reducing later cognitive load or stringent timing constraints on consecutive actions. This measure was shown to account for almost as much variance in performance in a generalized estimating equation analysis as the other three more straightforward predictors together.

Our study demonstrates that considerable differences in the divided attention ability of normal subjects can be identified with minimal efforts, using a small sample and applying a relatively short period of practice. The results of this work also support the assumption that performance in complex and challenging environments is determined in large part by the strategies employed by individuals. Since explorations may be revealed by means of measuring gaze patterns, such experiments may provide falsifying evidences for our arguments.
