# Correction: Strategic predictors of performance in a divided attention task

**DOI:** 10.1371/journal.pone.0197309

**Published:** 2018-05-10

**Authors:** Róbert Adrian Rill, Kinga Bettina Faragó, András Lőrincz

Information is missing from the Funding section. The complete, correct funding information is as follows: The research was carried out as part of the EITKIC 12-1-2012-0001 project, which is supported by the Hungarian Government, managed by the National Development Agency, financed by the Research and Technology Innovation Fund and was performed in cooperation with the EIT Digital Budapest Associate Partner Group (www.budapestictnetwork.elte.hu). The research has also been supported by the European Union, co-financed by the European Social Fund (EFOP-3.6.2-16-2017-00013). The funders had no role in study design, data collection and analysis, decision to publish, or preparation of the manuscript.
